# Machine learning-based prediction of hypoglycemia severity in hospitalized diabetic patients

**DOI:** 10.3389/fendo.2025.1634358

**Published:** 2025-09-17

**Authors:** Hongjian Jia, Jietao Zhang

**Affiliations:** Department of General Practice, The Affiliated Hospital of Qingdao University, Qingdao, China

**Keywords:** type 2 diabetes mellitus, hypoglycemia, risk prediction, machine learning, clinical analysis

## Abstract

**Objective:**

To identify risk factors for hypoglycemia in hospitalized patients with type 2 diabetes mellitus (T2DM) and develop predictive models for hypoglycemia severity based on machine learning algorithms.

**Methods:**

Adult non-pregnant hospitalized patients diagnosed with T2DM were retrospectively enrolled from the electronic medical record system of the Affiliated Hospital of Qingdao University. Patients were categorized into hypoglycemia groups (mild, moderate-to-severe) or a non-hypoglycemia group based on inpatient venous plasma glucose levels. After data preprocessing, univariate and multivariate analyses were conducted to identify significant predictors. Three predictive models (XGBoost, Random Forest [RF], and Logistic Regression) were subsequently constructed and validated to evaluate their predictive performances.

**Results:**

From an initial cohort of 8,947 patients, 1,798 patients were included after data screening. Among the evaluated models, the RF model demonstrated the highest predictive accuracy (93.3%) and Kappa coefficient (0.873), followed by XGBoost (accuracy: 92.6%, Kappa: 0.860). Logistic regression exhibited comparatively lower performance (accuracy: 83.8%, Kappa: 0.685). The macro-average area under the ROC curve (AUC) values for RF, XGBoost, and logistic regression were 0.960, 0.955, and 0.788, respectively, highlighting the superior discriminative capability of the RF model. While both XGBoost and RF models identified glycemic control metrics and glucose variability as core predictors for hypoglycemia, the RF model additionally emphasized medication usage, whereas XGBoost prioritized basal metabolic parameters.

**Conclusions:**

The RF model outperformed XGBoost and conventional logistic regression in predicting hypoglycemia severity among hospitalized T2DM patients. The results emphasize the importance of closely monitoring glucose levels and glucose variability during diabetes management to prevent hypoglycemia. The developed model provides a foundation for implementing preventive strategies to reduce hypoglycemia occurrence in hospitalized patients with T2DM.

## Introduction

Diabetes mellitus (DM) is a major chronic disease worldwide, with prevalence rates steadily rising over recent decades (1). Diabetes and its associated complications pose significant threats to patient health and quality of life, greatly impacting patients’ daily activities and potentially leading to mortality in severe cases. Among these complications, hypoglycemia is particularly critical to prevent and manage due to its frequent occurrence and substantial health risks for diabetic patients (2). Hypoglycemic events in diabetic patients are multifactorial, influenced by medications, diet, lifestyle, and comorbidities (3–5). Recently, stringent glycemic control strategies have been associated with an increased risk of hypoglycemia. However, hypoglycemia can undermine the long-term benefits gained from good glycemic management, underscoring the importance of carefully balancing the benefits and risks of intensive glucose management.

Hospitals play a central role in glycemic control, adjustments of antidiabetic medications, and individualized care for diabetic patients, placing heightened responsibilities on healthcare professionals. Patients with diabetes commonly exhibit multiple comorbidities that contribute independently to hypoglycemia risk. Studies have identified older age, renal impairment, liver dysfunction, poor nutritional status, inappropriate medication use, and debilitating diseases as critical risk factors for hypoglycemia. Clinical practitioners thus must analyze these risk factors systematically to accurately assess the probability of hypoglycemia and implement effective preventive measures.

Predicting hypoglycemia accurately, however, remains challenging due to the complex interplay of various clinical and biological factors. Although multiple studies have confirmed insulin therapy (6), impaired renal function, and suboptimal glycemic control as significant predictors of hypoglycemia, reliably forecasting such events remains difficult. Traditional logistic regression models have been widely employed to identify risk factors but are limited by their assumption of linear relationships between predictors and outcomes.

Given the limitations of traditional statistical methods in capturing complex clinical relationships, this study was motivated to explore advanced machine learning algorithms for predicting hypoglycemia severity in hospitalized T2DM patients. Thus, the current study aimed to develop and systematically compare three predictive models—multinomial logistic regression, XGBoost, and RF—to identify independent risk factors and predict hypoglycemia severity. We further conducted a feature importance analysis to highlight clinically significant predictors of hypoglycemia risk. This comparative approach is expected to improve early clinical intervention and personalized management. The paper is structured as follows: the methods section describes the study population, data collection, and model development; the results section presents patient characteristics and model performances; the discussion elaborates clinical implications and comparative insights from the modeling results; finally, limitations and future research directions are provided.

## Literature review

Hypoglycemia remains a significant challenge in managing hospitalized patients with type 2 diabetes mellitus (T2DM), often resulting from complex interactions among clinical factors such as medication usage, renal function, and glycemic variability. Traditionally, logistic regression models have been widely employed to identify predictors of hypoglycemia risk due to their interpretability and simplicity. However, these models assume linear relationships and may not effectively capture intricate interactions among clinical variables, limiting their predictive capabilities in clinical practice.

With advancements in machine learning techniques, models such as Extreme Gradient Boosting (XGBoost) and Random Forest (RF) have shown remarkable performance in predicting complex clinical outcomes due to their ability to capture nonlinear interactions and handle high-dimensional data. Several previous studies have explored machine learning approaches to predict hypoglycemia in diabetic patients. For instance, Melih Agraz et al. used multi-view collaborative training of machine learning models on imbalanced datasets to improve hypoglycemia prediction (7). Harald Witte et al. used XGBoost alone to train models related to glycemic decompensation (8). Christopher Duckworth et al. also used XGBoost alone to predict glycemic decompensation (hyperglycemia and hypoglycemia) (9). Mai Shi et al. developed a multidimensional ML model based on electronic health records (EHR) to predict hypoglycemia in the elderly population (10). However, most of these studies have focused solely on hypoglycemia or hyperglycemia, lacking analysis of the severity of hypoglycemia. Additionally, few studies have used real hospital patient data to comprehensively compare multiple models. This study addresses this shortcoming by evaluating logistic regression, random forest, and XGBoost models to perform multi-class classification of hypoglycemia severity using clinical variables.

## Methods

### Study design and population

This retrospective study was conducted using data from the electronic medical record system of the Affiliated Hospital of Qingdao University. Initially, 8,947 adult, non-pregnant hospitalized patients with a confirmed diagnosis of T2DM were identified. After applying inclusion and exclusion criteria, a total of 1,798 patients were included in the final analysis.

### Data collection

Clinical and demographic data were extracted from electronic medical records, including demographic characteristics, clinical features, laboratory findings, and antidiabetic medication use. Variables collected were age, gender, body mass index (BMI) classification, Charlson Comorbidity Index (CCI), glycated hemoglobin (HbA1c), mean blood glucose levels, serum creatinine, C-peptide, lipid profile, and use of antidiabetic medications (e.g., insulin, metformin, DPP - 4 inhibitors).

### Patient grouping

Patients were categorized into three groups based on venous plasma glucose levels measured during hospitalization:

- Normal glycemia: >3.9 mmol/L
- Mild hypoglycemia: 3.0 – 3.9 mmol/L
- Moderate-to-severe hypoglycemia: <3.0 mmol/L

### Statistical analysis

Univariate analyses were conducted using the chi-square test for categorical variables and the Kruskal-Wallis test for continuous variables. Variables with a P-value <0.05 were entered into a multivariate multinomial logistic regression analysis. Model performance was evaluated using overall accuracy, Kappa statistic, the area under the receiver operating characteristic (ROC) curve (AUC), and confusion matrices. All statistical analyses were performed using R software and SPSS.

### Machine learning model development

Three predictive models were developed and evaluated: multinomial logistic regression, XGBoost, and Random Forest. Hyperparameters for the XGBoost and RF models were optimized using cross-validation. Multiclass ROC curves were constructed using a One-vs-Rest approach to visualize and compare the models’ predictive capabilities.

No independent testing set was separated in this study. Model development and evaluation were entirely conducted using 5-fold cross-validation on the full dataset. During training, hyperparameters of XGBoost and Random Forest were optimized within each fold using grid search. For XGBoost, the number of boosting rounds (nrounds), tree depth (max_depth), and learning rate (eta) were tuned. For Random Forest, the number of trees (ntree) and the number of variables randomly selected at each split (mtry) were adjusted. The best parameter set in each case was selected based on the highest average accuracy and Kappa coefficient across the cross-validation folds. Final performance metrics, including accuracy, Kappa coefficient, and area under the ROC curve (AUC), represent the average results across the cross-validation folds. This strategy ensured internal validation while minimizing overfitting and allowing fair comparison across models.

### Model performance evaluation

Model performance evaluation was based on three key metrics: overall accuracy, Cohen’s Kappa coefficient, and area under the receiver operating characteristic curve (AUC). These metrics were calculated for each fold and averaged to obtain the final performance results. Additionally, multiclass ROC curves were generated using a One-vs-Rest approach to evaluate model discrimination across the three hypoglycemia severity classes.

## Results

### Patient characteristics

A total of 1,798 hospitalized diabetic patients were included in the final analysis. Based on inpatient blood glucose measurements, patients were divided into three groups: normoglycemic, mild hypoglycemia, and moderate-to-severe hypoglycemia. Significant differences were observed among the three groups in age, Charlson Comorbidity Index (CCI), the number of glucose-lowering medication classes, triglycerides (TG), serum creatinine, glycated hemoglobin (HbA1c), C-peptide, mean glucose levels, cholesterol, gender distribution, BMI classification, and use of various glucose-lowering medications (including SGLT2 inhibitors, α-glucosidase inhibitors, metformin, thiazolidinediones, and insulin) (all P < 0.05). Baseline characteristics of the study population are detailed in **Table 1**.

**Table 1:** Baseline characteristics of patients by hypoglycemia severity group.

Variables	Total (n = 1798)	Normal (n = 1046)	Mild hypoglycemia (n = 593)	Moderate-to-severe hypoglycemia (n = 159)	P
Age, M (Q_1_, Q_3_)	62.00 (54.00, 68.00)	61.00 (54.00,67.00)	63.00 (54.00,69.00)	62.00 (56.00,71.00)	**0.012**
Charlson, M (Q_1_, Q_3_)	1.00 (1.00, 1.00)	1.00 (1.00,1.00)	1.00 (1.00,3.00)	2.00 (1.00,3.00)	**<.001**
DrugClassCount, M (Q_1_, Q_3_)	2.00 (1.00, 3.00)	2.00 (1.00,3.00)	3.00 (2.00,3.00)	2.00 (1.00,3.00)	**<.001**
TG, M (Q_1_, Q_3_)	1.10 (0.77, 1.67)	1.15 (0.79,1.75)	1.04 (0.75,1.48)	1.02 (0.68,1.63)	**<.001**
Creatinine, M (Q_1_, Q_3_)	76.60 (57.00, 93.00)	73.00 (55.00,90.25)	79.50 (59.00,97.00)	90.00 (64.80,133.22)	**<.001**
HbA1c, M (Q_1_, Q_3_)	6.20 (5.90, 7.30)	6.10 (5.80,6.30)	7.70 (6.50,9.50)	7.60 (6.50,9.45)	**<.001**
C Peptide, M (Q_1_, Q_3_)	1.67 (0.70, 2.67)	2.10 (1.45,3.04)	0.69 (0.33,1.63)	0.73 (0.24,2.00)	**<.001**
GlucoseAvg, M (Q_1_, Q_3_)	4.92 (3.84, 5.63)	5.19 (4.78,5.65)	3.73 (3.48,4.45)	4.77 (2.79,8.49)	**<.001**
Cholesterol, M (Q_1_, Q_3_)	4.25 (3.42, 5.03)	4.21 (3.39,4.95)	4.35 (3.51,5.28)	4.06 (3.28,4.98)	**0.005**
Sex, n(%)					**0.014**
M	999 (55.56)	609 (58.22)	301 (50.76)	89 (55.97)	
W	799 (44.44)	437 (41.78)	292 (49.24)	70 (44.03)	
BMI Class, n(%)					**<.001**
Overweight	708 (39.38)	439 (41.97)	227 (38.28)	42 (26.42)	
Obese	339 (18.85)	232 (22.18)	88 (14.84)	19 (11.95)	
Underweight	49 (2.73)	14 (1.34)	22 (3.71)	13 (8.18)	
Normal	702 (39.04)	361 (34.51)	256 (43.17)	85 (53.46)	
DPP4, n(%)					0.869
Not used	1063 (59.12)	615 (58.80)	351 (59.19)	97 (61.01)	
Used	735 (40.88)	431 (41.20)	242 (40.81)	62 (38.99)	
GLP1, n(%)					0.056
Not used	1753 (97.50)	1012 (96.75)	584 (98.48)	157 (98.74)	
Used	45 (2.50)	34 (3.25)	9 (1.52)	2 (1.26)	
SGLT2, n(%)					**0.027**
Not used	1560 (86.76)	894 (85.47)	518 (87.35)	148 (93.08)	
Used	238 (13.24)	152 (14.53)	75 (12.65)	11 (6.92)	
AGI, n(%)					**<.001**
Not used	796 (44.27)	519 (49.62)	214 (36.09)	63 (39.62)	
Used	1002 (55.73)	527 (50.38)	379 (63.91)	96 (60.38)	
Metformin, n(%)					**<.001**
Not used	740 (41.16)	346 (33.08)	298 (50.25)	96 (60.38)	
Used	1058 (58.84)	700 (66.92)	295 (49.75)	63 (39.62)	
TZD, n(%)					**0.013**
Not used	1700 (94.55)	975 (93.21)	571 (96.29)	154 (96.86)	
Used	98 (5.45)	71 (6.79)	22 (3.71)	5 (3.14)	
Insulin, n(%)					**<.001**
Not used	931 (51.78)	811 (77.53)	96 (16.19)	24 (15.09)	
Used	867 (48.22)	235 (22.47)	497 (83.81)	135 (84.91)	

Values highlighted in red indicate statistical significance (P < 0.05). Values in black indicate no significant difference.

**Figure 1:**
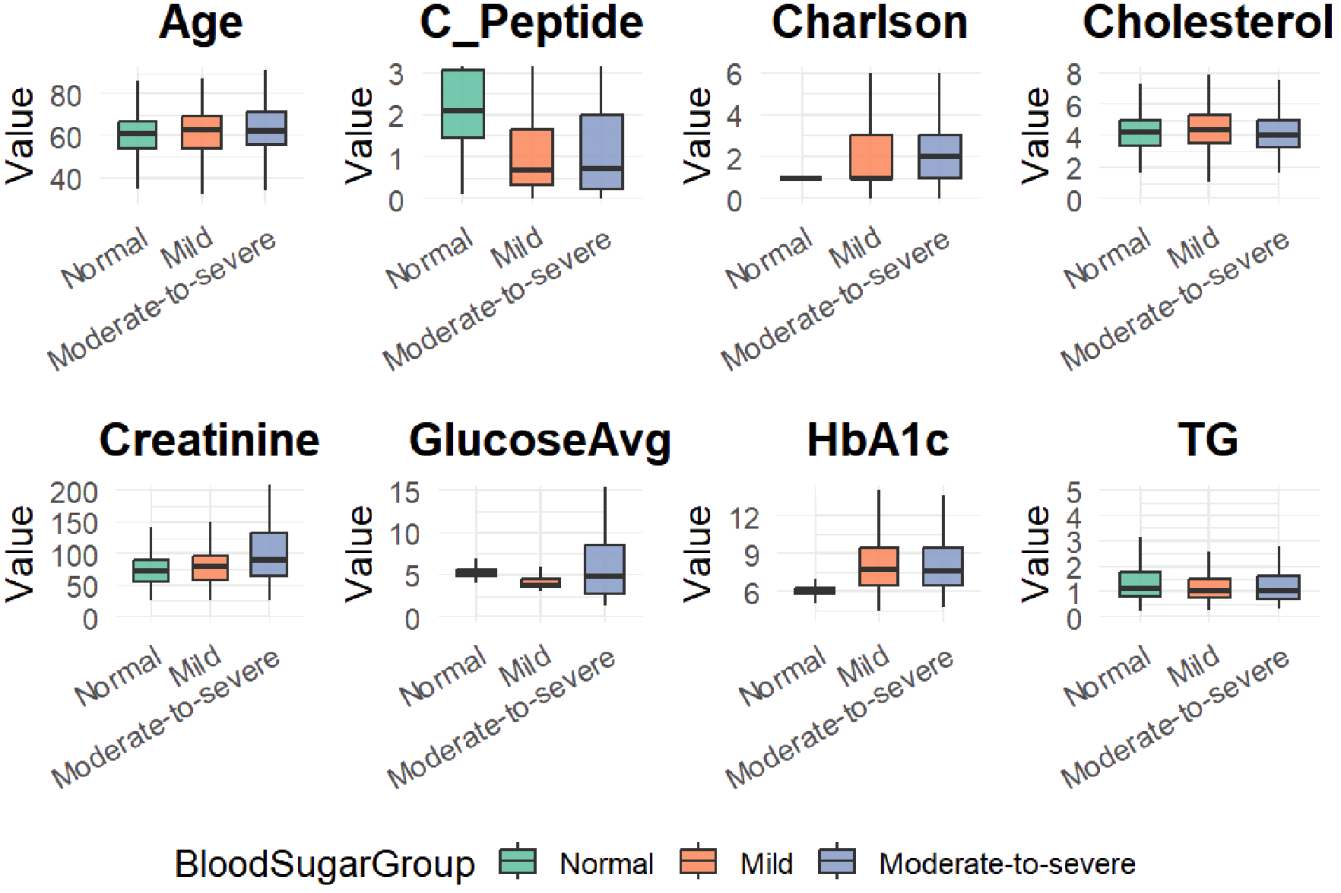
Boxplots of eight clinical variables across hypoglycemia severity groups (Normal, Mild, and Moderate-to-severe).

To visualize the distributional characteristics of numeric clinical variables across hypoglycemia severity groups, boxplots were generated (**Figure 1**). These plots provide an intuitive depiction of intergroup variability and outliers, complementing the statistical summary presented in **Table 1**. Categorical variables were not included in this visualization due to the nature of boxplots being suited for continuous variables only.

### Univariate and Multivariate Logistic Regression Analyses

In the univariate logistic regression analysis (**Table 2**), gender, Charlson comorbidity index, number of glucose-lowering medication classes, BMI classification, triglycerides (TG), serum creatinine, HbA1c, C-peptide, mean glucose levels, cholesterol, and use of specific hypoglycemic drugs (metformin, α-glucosidase inhibitors, insulin) were significantly associated with the risk of hypoglycemia (P < 0.05). Specifically, an increased Charlson index, lower BMI, reduced C-peptide levels, decreased TG, elevated HbA1c, elevated creatinine levels, and insulin use were linked to a higher risk of hypoglycemia.

**Table 2:** Univariate multinomial logistic regression analysis for predictors of hypoglycemia severity.

Variables	Mild hypoglycemia vs normal (OR [95%CI])	P value	Moderate-to-severe hypoglycemia vs normal (OR [95%CI])	P value
Sex (Male vs Female)	0.74 (0.60 – 0.91)	**0.004**	0.91 (0.65 – 1.28)	0.593
Age (per year)	1.01 (1.00 – 1.02)	0.156	1.01 (1.00 – 1.03)	0.133
Charlson score	3.81 (3.18 – 4.56)	**<0.001**	4.07 (3.35 – 4.94)	**<0.001**
Drug Class Count	1.59 (1.44 – 1.75)	**<0.001**	1.28 (1.09 – 1.51)	**0.002**
BMI Overweight vs Normal	0.73 (0.58 – 0.91)	**0.006**	0.41 (0.27 – 0.60)	**<0.001**
BMI Obese vs Normal	0.54 (0.40 – 0.72)	**<0.001**	0.35 (0.21 – 0.59)	**<0.001**
BMI Underweight vs Normal	2.22 (1.11 – 4.41)	**0.024**	3.94 (1.79 – 8.70)	**0.001**
TG (mmol/L)	0.85 (0.77 – 0.94)	**0.002**	0.95 (0.83 – 1.09)	0.454
HbA1c (%)	10.15 (7.76 – 13.27)	**<0.001**	10.07 (7.65 – 13.26)	**<0.001**
Creatinine (umol/L)	1.005 (1.003 – 1.007)	**<0.001**	1.006 (1.004 – 1.008)	**<0.001**
GlucoseAvg (mmol/L)	0.77 (0.72 – 0.83)	**<0.001**	1.11 (1.04 – 1.18)	**0.003**
C-Peptide (ng/mL)	0.68 (0.63 – 0.74)	**<0.001**	0.87 (0.80 – 0.95)	**0.002**
Cholesterol (mmol/L)	1.15 (1.07 – 1.23)	**<0.001**	0.99 (0.88 – 1.11)	0.811
DPP4i (No vs Yes)	1.02 (0.83 – 1.25)	0.876	1.10 (0.78 – 1.54)	0.597
GLP1-RA (No vs Yes)	2.18 (1.04 – 4.58)	**0.039**	2.64 (0.63 – 11.09)	0.186
SGLT2i (No vs Yes)	1.17 (0.87 – 1.58)	0.289	2.29 (1.21 – 4.32)	**0.011**
AGI (No vs Yes)	0.57 (0.47 – 0.70)	**<0.001**	0.67 (0.47 – 0.94)	**0.019**
Metformin (No vs Yes)	2.04 (1.66 – 2.51)	**<0.001**	3.08 (2.19 – 4.34)	**<0.001**
TZD (No vs Yes)	1.89 (1.16 – 3.08)	**0.011**	2.24 (0.89 – 5.64)	0.086
Insulin (No vs Yes)	0.06 (0.04 – 0.07)	**<0.001**	0.05 (0.03 – 0.08)	**<0.001**

Values highlighted in red indicate statistical significance (P < 0.05). Values in black indicate no significant difference.

In the multivariate multinomial logistic regression analysis (**Table 3**), independent factors associated with mild hypoglycemia included older age (OR = 0.966, 95% CI: 0.951 – 0.981, P < 0.001), higher Charlson comorbidity index (OR = 2.684, 95% CI: 2.121 – 3.398, P < 0.001), increased creatinine (OR = 1.005, 95% CI: 1.003 – 1.007, P < 0.001), elevated HbA1c (OR = 10.570, 95% CI: 7.554 – 14.789, P < 0.001), lower mean glucose levels (OR = 0.545, 95% CI: 0.479 – 0.619, P < 0.001), and insulin use (OR = 0.205, 95% CI: 0.127 – 0.332, P < 0.001).

**Table 3:** Multivariate multinomial logistic regression results for mild and moderate-to-severe hypoglycemia.

Variables	Mild hypoglycemia vs normal (OR [95%CI])	P value	Moderate-to-severe hypoglycemia vs normal (OR [95%CI])	P value
Age	0.966 (0.951 – 0.981)	**<0.001**	0.973 (0.954 – 0.993)	**0.007**
Charlson Score	2.684 (2.121 – 3.398)	**<0.001**	2.744 (2.141 – 3.517)	**<0.001**
Drug Class Count	0.788 (0.571 – 1.086)	0.146	0.791 (0.520 – 1.202)	0.272
TG	0.857 (0.712 – 1.033)	0.105	0.904 (0.717 – 1.139)	0.391
Creatinine	1.005 (1.003 – 1.007)	**<0.001**	1.005 (1.003 – 1.007)	**<0.001**
HbA1c	10.570 (7.554 – 14.789)	**<0.001**	10.047 (7.127 – 14.163)	**<0.001**
C-Peptide	1.023 (0.945 – 1.108)	0.570	1.092 (0.994 – 1.200)	0.067
Glucose Avg	0.545 (0.479 – 0.619)	**<0.001**	0.633 (0.554 – 0.723)	**<0.001**
Cholesterol	1.021 (0.894 – 1.166)	0.758	0.856 (0.728 – 1.006)	0.059
Sex (Male)	0.995 (0.692 – 1.431)	0.980	1.086 (0.682 – 1.730)	0.728
SGLT2 Not Used	0.953 (0.500 – 1.817)	0.883	1.809 (0.714 – 4.580)	0.211
AGI Not Used	0.680 (0.425 – 1.089)	0.109	0.680 (0.362 – 1.277)	0.230
Metformin Not Used	1.034 (0.628 – 1.701)	0.895	1.162 (0.607 – 2.223)	0.651
TZD Not Used	1.640 (0.673 – 3.999)	0.277	1.601 (0.475 – 5.390)	0.447
Insulin Not Used	0.205 (0.127 – 0.332)	**<0.001**	0.170 (0.086 – 0.336)	**<0.001**
BMI Class - Overweight	1.043 (0.699 – 1.557)	0.836	0.511 (0.302 – 0.865)	**0.012**
BMI Class - Obese	1.254 (0.765 – 2.057)	0.370	0.747 (0.380 – 1.470)	0.399
BMI Class - Underweight	1.964 (0.600 – 6.427)	0.265	3.654 (1.045 – 12.781)	**0.043**

Values highlighted in red indicate statistical significance (P < 0.05). Values in black indicate no significant difference.

For moderate-to-severe hypoglycemia, independent predictors included older age (OR = 0.973, 95% CI: 0.954 – 0.993, P = 0.007), elevated Charlson comorbidity index (OR = 2.744, 95% CI: 2.141 – 3.517, P < 0.001), increased serum creatinine (OR = 1.005, 95% CI: 1.003 – 1.007, P < 0.001), elevated HbA1c (OR = 10.047, 95% CI: 7.127 – 14.163, P < 0.001), lower mean glucose levels (OR = 0.633, 95% CI: 0.554 – 0.723, P < 0.001), and insulin use (OR = 0.170, 95% CI: 0.086 – 0.336, P < 0.001). Additionally, overweight status was a protective factor against moderate-to-severe hypoglycemia (OR = 0.511, 95% CI: 0.302 – 0.865, P = 0.012), while underweight status significantly increased the risk (OR = 3.654, 95% CI: 1.045 – 12.781, P = 0.043).

Overall, the multivariate logistic regression model demonstrated good fit (χ² = 1585.430, df = 40, P < 0.001; Cox-Snell R² = 0.586; Nagelkerke R² = 0.703; McFadden R² = 0.492).

### Machine learning model evaluation

**Figure 2:**
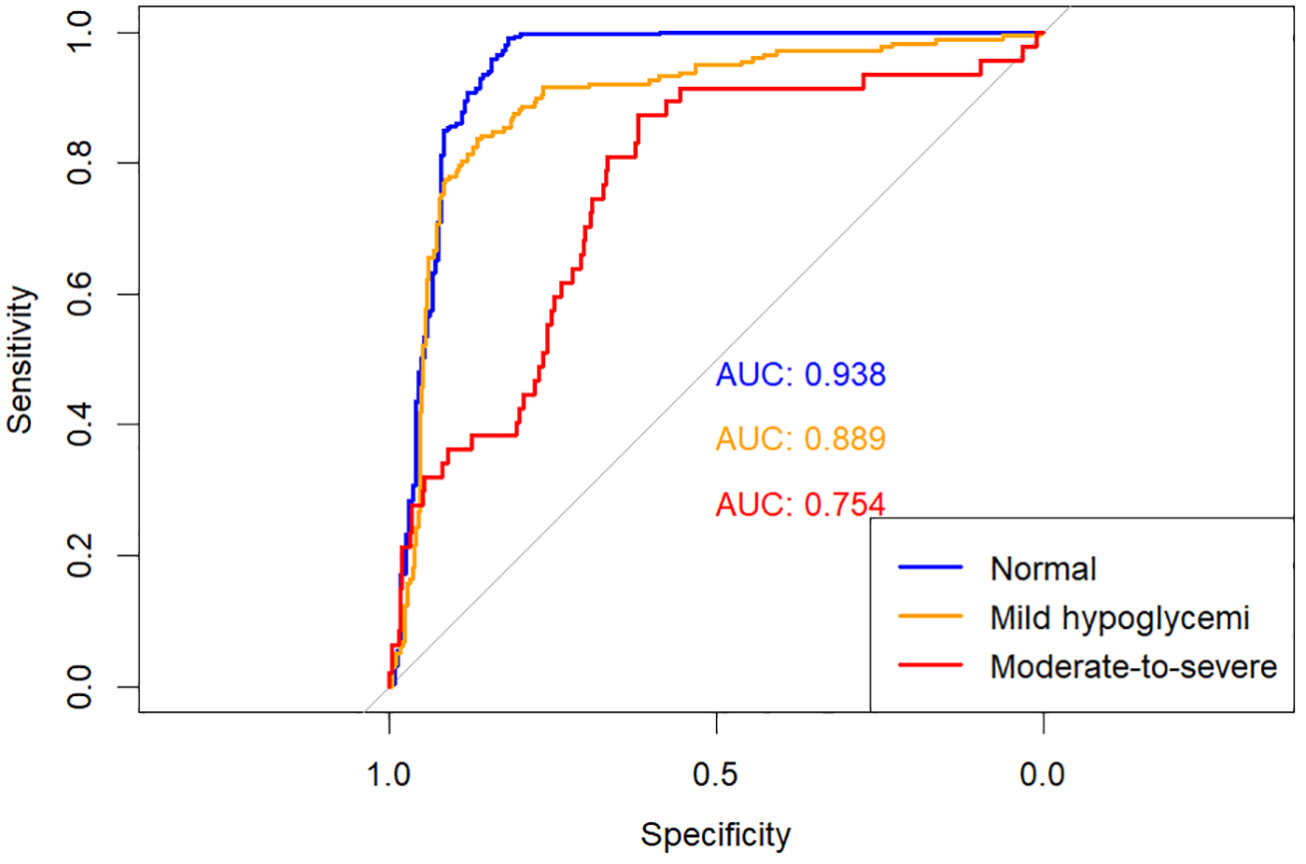
One-vs-rest ROC curves for multinomial logistic regression model.

**Figure 3:**
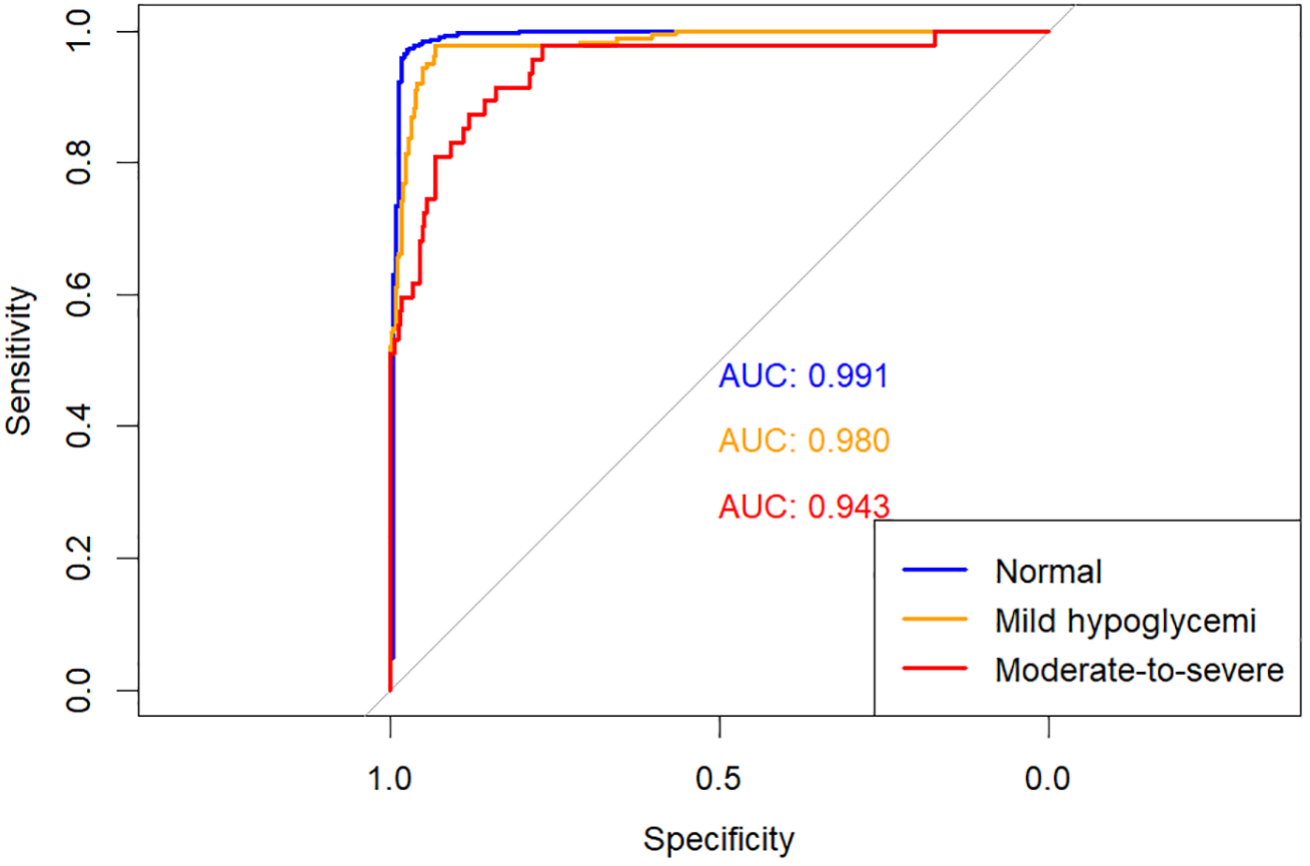
One-vs-rest ROC curves for the XGBoost model.

**Figure 4:**
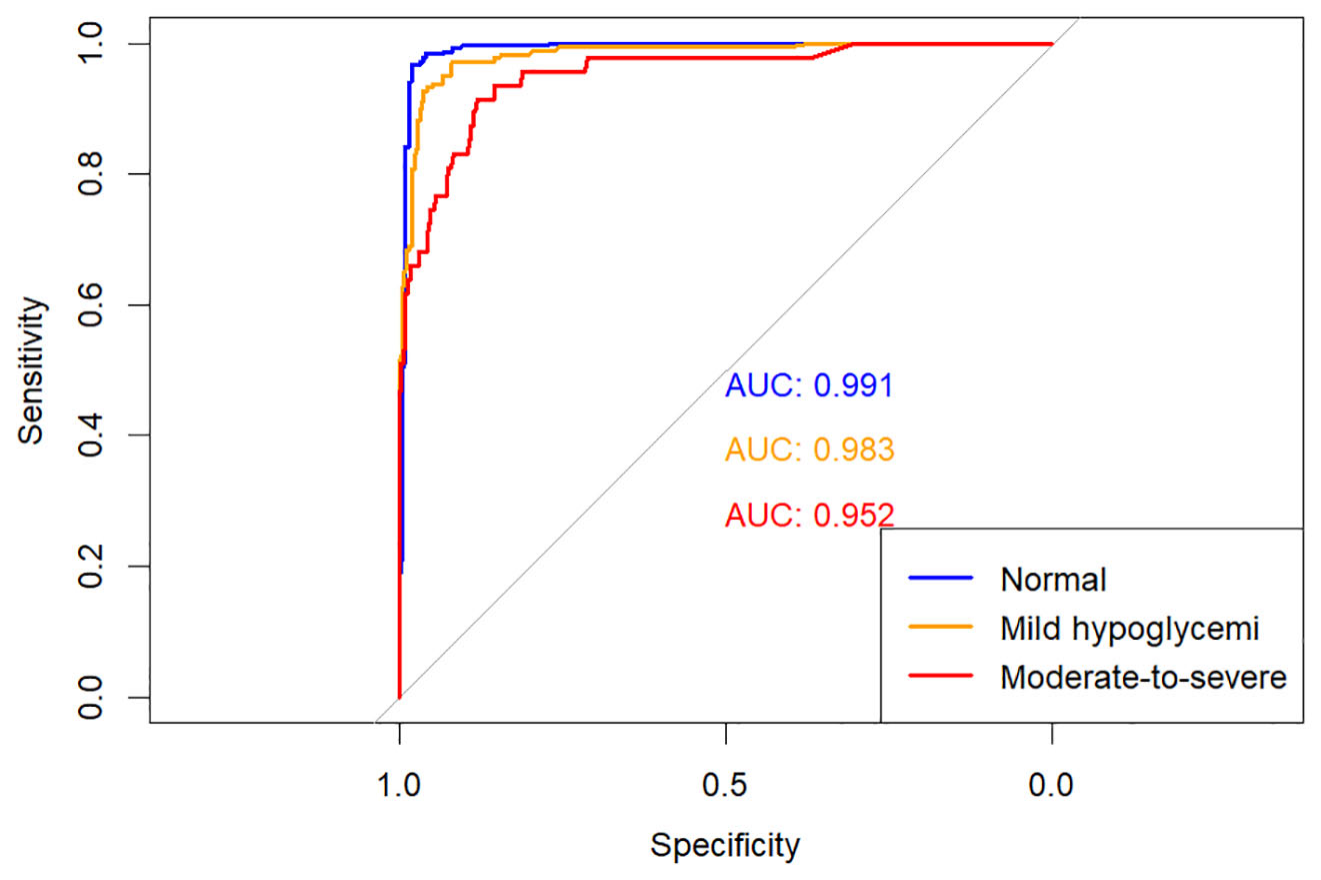
One-vs-rest ROC curves for random forest model.

**Figure 5:**
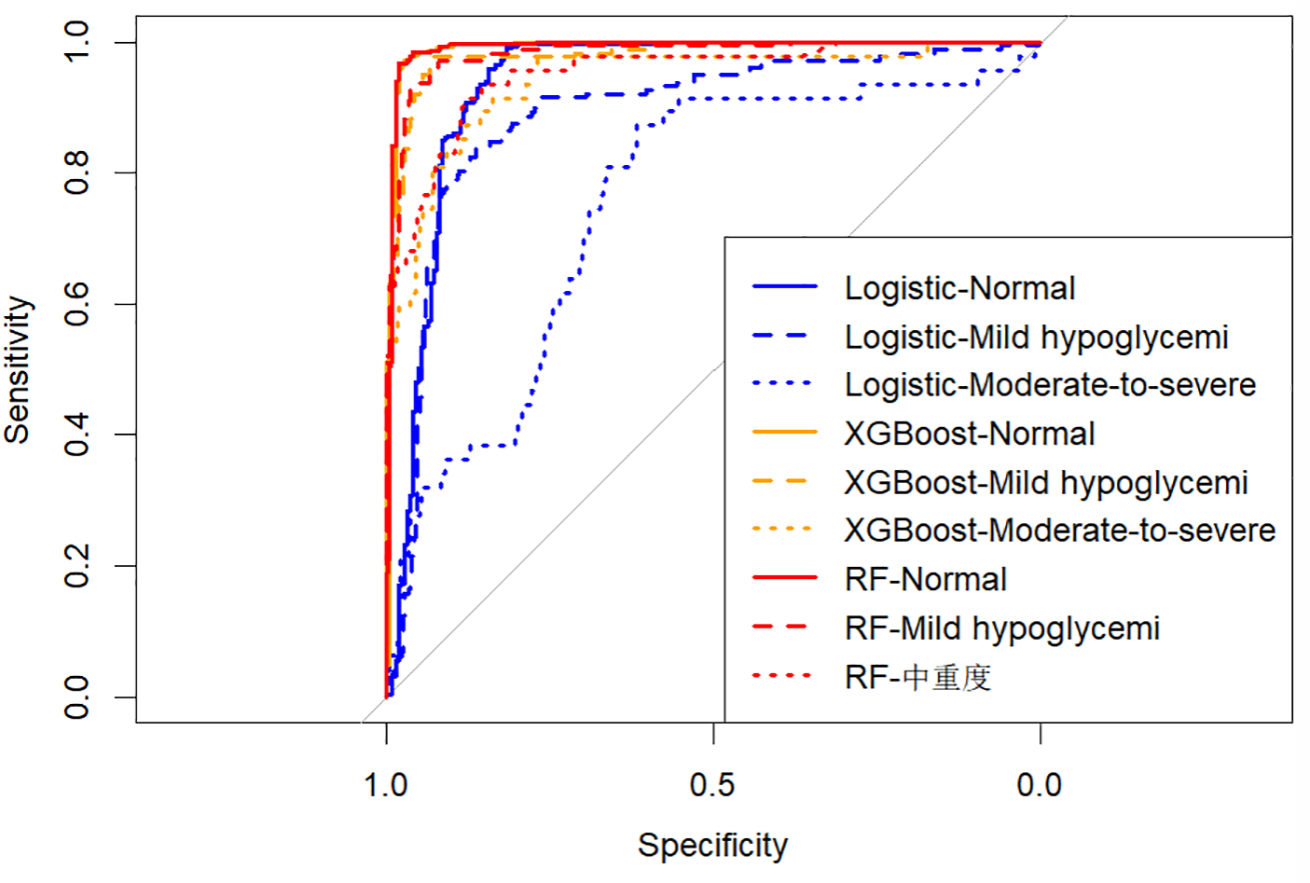
Comparison of ROC curves across logistic regression, XGBoost, and random forest models.

The predictive performances of multinomial logistic regression, XGBoost, and random forest (RF) models were evaluated using receiver operating characteristic (ROC) curves constructed through a One-vs-Rest approach. ROC curves for each model are illustrated individually in **Figures 2**–**4**, and a direct comparative analysis among the three models is presented in **Figure 5**.

The multinomial logistic regression model yielded area under the curve (AUC) values of 0.938 for predicting normoglycemia, 0.889 for mild hypoglycemia, and 0.754 for moderate-to-severe hypoglycemia. The XGBoost model demonstrated notably higher AUCs of 0.991, 0.980, and 0.943, respectively. Similarly, the RF model achieved AUCs of 0.991, 0.983, and 0.952 for the three respective severity categories.

Comparative ROC analysis (**Figure 5**) clearly indicated superior discriminative performance of both XGBoost and RF models across all hypoglycemia severity categories compared to logistic regression.

### Model performance comparison

To comprehensively evaluate predictive performances, we compared the overall accuracy, Kappa coefficient, and macro-average AUC of the three models (**Table 4**). The RF model achieved the highest accuracy (93.3%) and Kappa coefficient (0.873), followed closely by XGBoost (accuracy: 92.6%, Kappa: 0.860). Logistic regression demonstrated relatively lower performance, with an accuracy of 83.8% and Kappa of 0.685. Consistently, the macro-average AUC values for RF, XGBoost, and logistic regression were 0.960, 0.955, and 0.788, respectively, further emphasizing the superior predictive capability of the tree-based machine learning algorithms.

**Table 4:** Performance comparison of three models: Accuracy, AUC, and Kappa.

Model	Accuracy	Kappa	Macro AUC
Logistic Regression	0.838	0.6845	0.7878
XGBoost	0.9255	0.8603	0.9553
Random Forest	0.9330	0.8729	0.9596

**Figure 6:**
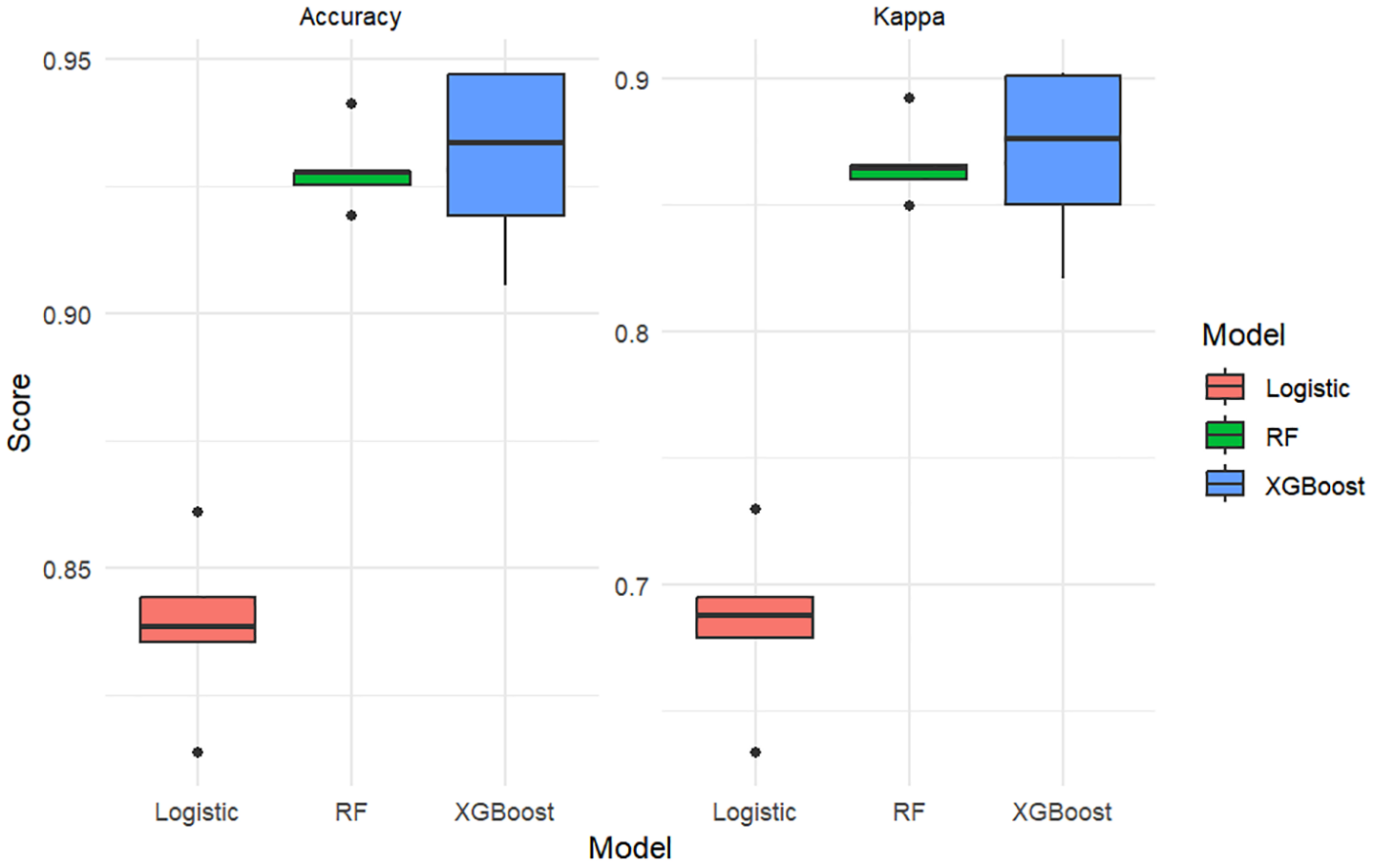
Boxplots comparing model performance (Accuracy and Kappa coefficient) across three classifiers using 5-fold cross-validation.

Note: The boxplot metrics are derived from 5-fold cross-validation, while **Table 4** reports final model performance on evaluation sets.

To further evaluate the consistency and robustness of model performance, we applied 5-fold cross-validation and visualized the distribution of prediction accuracy and Kappa coefficient for each model (**Figure 6**). The Random Forest model consistently demonstrated the highest predictive performance (mean accuracy ≈ 93.2%, Kappa ≈ 0.872), followed closely by XGBoost (accuracy ≈ 92.6%, Kappa ≈ 0.860). Logistic regression showed comparatively lower performance (accuracy ≈ 84.0%, Kappa ≈ 0.682). These values were consistent with the results in **Table 4**, confirming the superiority of tree-based models in this context.

### Feature importance analysis

**Figure 7:**
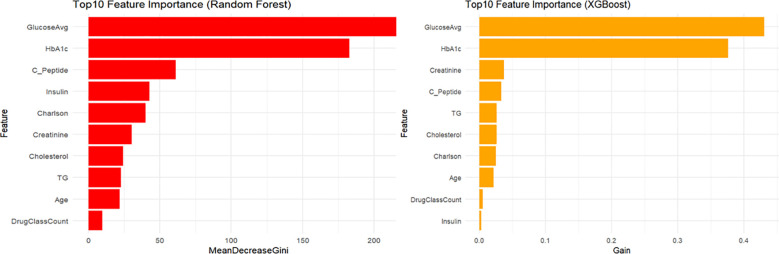
Top 10 feature importance.

Feature importance analysis (**Figure 7**) identified mean blood glucose level and HbA1c as the two most critical predictors in both XGBoost and RF models. Subsequent important predictors in the XGBoost model included creatinine, C-peptide, and triglycerides (TG). In the RF model, following mean glucose and HbA1c, important predictors included C-peptide, insulin usage, and the Charlson comorbidity index.

## Discussion

In the present study, we developed and systematically compared three predictive models—multinomial logistic regression, XGBoost, and random forest (RF)—to identify key predictors of hypoglycemia severity among hospitalized patients with diabetes. Our results demonstrated that both machine learning models (XGBoost and RF) exhibited superior discriminative performance compared to traditional logistic regression, especially for detecting moderate-to-severe hypoglycemia, this has some modeling studies with the same results (11). We utilized the One-vs-Rest strategy, a standard and effective approach for generating ROC curves in multiclass classification problems, allowing robust evaluation and comparison of each model’s predictive performance across hypoglycemia categories.

As detailed in **Table 4**, the random forest model demonstrated the best overall predictive performance, with the highest accuracy (93.3%), Kappa coefficient (0.873), and macro-average AUC (0.960). These values reflect excellent classification agreement and discrimination for hypoglycemia severity prediction. The XGBoost model showed slightly lower but still robust performance (accuracy 92.6%, Kappa 0.860, AUC 0.955), confirming its strength in handling complex nonlinear data. In contrast, the logistic regression model yielded lower accuracy (83.8%) and Kappa (0.685), along with a substantially lower AUC (0.788), possibly due to its linear assumptions and inability to capture complex feature interactions. These findings highlight the superior performance of ensemble learning models in identifying subtle patterns and interactions in clinical data, and support their potential value in hypoglycemia risk stratification.

The dataset used to construct our predictive models comprised common clinical and laboratory variables routinely available for hospitalized patients with type 2 diabetes mellitus (T2DM). The logistic regression model identified seven statistically significant predictors (P < 0.05), while both XGBoost and RF models highlighted the top 10 most influential variables. Notably, six predictors—age, Charlson Comorbidity Index (CCI), serum creatinine, HbA1c, mean glucose levels, and insulin use—emerged consistently as significant factors across all three models. A number of previous modeling studies and factor analysis studies have similarly given the same conclusions for one or more of these factors (12–14). While previous studies have individually confirmed the significance of one or several of these factors, our research uniquely integrated multiple models for comprehensive comparison, offering a broader perspective than single-model studies previously reported.

A key advantage of our study lies in its reliance on routinely collected clinical data. This facilitates rapid assessment of hypoglycemia risk without additional specialized testing, thus granting medical staff valuable time to implement preventive strategies and appropriate treatments.

Regarding predictive variables, HbA1c has consistently been identified as a crucial factor in hypoglycemia research. Prior studies have reported that both excessively high HbA1c levels (>9%) and overly stringent glycemic control (HbA1c <7%) are associated with increased hypoglycemia risk (15). Additionally, insulin use within these HbA1c ranges further exacerbates hypoglycemia risk. Both our univariate and multivariate analyses supported these findings, reinforcing that elevated HbA1c levels and insulin therapy are critical risk factors for hypoglycemia in diabetic patients.

In recent years, growing attention has focused on the association between the Charlson Comorbidity Index (CCI) and hypoglycemia. Most prior studies investigating comorbidities typically examined only one or a few components of the CCI, such as cardiovascular disease, renal impairment, or malignancy (16). These comorbid conditions have been associated with glucose instability and heightened risk of adverse glycemic events. Patients with multiple chronic diseases often experience altered drug metabolism, polypharmacy, and malnutrition, potentially interfering with glucose regulation and insulin sensitivity. Incorporating the CCI into all three predictive models in our study enhances its utility as a comprehensive clinical indicator. Unlike individual diagnoses, the CCI provides an aggregated measure of overall disease burden, capturing complex interactions between comorbidities and thereby improving the generalizability and interpretability of the predictive models. Integrating the CCI into predictive assessments may assist clinicians in accurately stratifying risk, particularly among elderly hospitalized patients or those with multiple chronic diseases, who inherently have higher susceptibility to hypoglycemic episodes (17). Early identification of high-CCI patients could facilitate personalized monitoring, medication adjustments, and tailored nutritional plans, thereby mitigating hypoglycemia risk.

Lastly, we conducted feature importance analyses for the top ten variables in the XGBoost and RF models. Both models consistently identified glycemic control and glucose variability as key predictors. Additionally, XGBoost emphasized basal metabolic parameters (e.g., creatinine and C-peptide), whereas the RF model placed greater emphasis on medication use. These findings highlight nuanced differences between machine learning approaches, underscoring their ability to provide targeted clinical insights into managing and preventing hypoglycemic events in hospitalized diabetic patients.

## Conclusions

In this study, we developed and compared three prediction models - multinomial logistic regression, XGBoost, and random forest - to identify factors associated with hypoglycemia severity in hospitalized diabetic patients. Our results showed that both machine learning models outperformed the traditional logistic regression model in terms of predictive performance, with higher accuracy and AUC values in all hypoglycemia categories. This time, the three models were designed with all commonly used clinical indicators, without the need for special tests, to prevent hypoglycemia as early as possible in type 2 diabetic patients and to avoid the adverse consequences caused by hypoglycemia. In a comparison of the three models and the focus of the models, the six variables of age, Charlson comorbidity index, creatinine, glycosylated hemoglobin, mean blood glucose level, and insulin use were consistently identified as core predictors in almost all models (7, 18, 19), This further enhances their clinical relevance. Future studies should focus on the prospective validation and practical application of these models to assess their clinical utility and impact on patient safety.

## Strengths and limitations

This study has several strengths. First, it comprehensively compared the performance of traditional and ensemble machine learning models in predicting hypoglycemia severity, highlighting the superior capability of tree-based algorithms. Second, it utilized a multiclass classification approach to stratify hypoglycemia into different severity levels, which has greater clinical relevance than binary classification. Third, model performance was rigorously evaluated using stratified 5-fold cross-validation, and visual comparisons were provided through ROC curves and boxplots, enhancing interpretability. Finally, the study was based on a large cohort of hospitalized patients with diabetes, offering real-world insights.

However, some limitations should be acknowledged. The data were derived from a single center, which may limit the generalizability of the findings. External validation using independent datasets from other institutions is needed. Additionally, the reliance on internal cross-validation without a separate test set may lead to optimistic performance estimates. Some potentially important predictors, such as postprandial glucose or nutritional interventions, were not available in the dataset. Moreover, the models have not yet been tested in real-time clinical settings, and their clinical utility remains to be validated.

## Data Availability

The datasets presented in this article are not readily available because The datasets generated and/or analyzed during the current study are not publicly available due to The Affiliated Hospital of Qingdao University but are available from the corresponding author on reasonable request. Requests to access the datasets should be directed to Hongjian Jia, jhj200018@163.com.
